# Towards the catalytic activation of inert small molecules by main-group ambiphiles

**DOI:** 10.1038/s42004-020-00371-4

**Published:** 2020-09-16

**Authors:** Rian D. Dewhurst, Marc-André Légaré, Holger Braunschweig

**Affiliations:** 1 Institute for Inorganic Chemistry, Julius-Maximilians-Universität Würzburg, Am Hubland, 97074 Würzburg Germany; 2 Institute for Sustainable Chemistry & Catalysis with Boron, Julius-Maximilians-Universität Würzburg, Am Hubland, 97074 Würzburg Germany; 3 Department of Chemistry, McGill University, 801 Sherbrooke St. West, Montréal, QC H3A 0B8 Canada

**Keywords:** Chemical bonding, Organometallic chemistry, Catalyst synthesis

## Abstract

The activation of very inert small molecules generally requires highly reactive activating species, but the high energy of these species makes their regeneration, and thus also catalytic turnover of the reaction, difficult to achieve. Here, the authors highlight the formidable challenge of overcoming the tradeoff between activating power and catalytic turnover in the context of main-group ambiphiles.

There is intense current interest in the possibility of using main-group elements in the place of transition metals (TMs) in molecular catalysis^1,2^, due mainly to the cost of the metals themselves and increasing concerns about the health and environmental impact of the residual metal retained in the products. Along these lines, main-group ambiphiles, as embodied by dual-site ambiphiles such as frustrated Lewis pairs (FLPs)^3^ and single-site ambiphiles such as singlet carbenes (CR_2_)^4–6^, constrained phosphines^7^, dicoordinate borylenes (LBR, L = Lewis donor)^8,9^ and dicoordinate group 14 cations^10^ have begun to emerge as powerful TM-free reagents for the activation of inert small molecules of catalytic interest (Fig. 1). Mimicking TMs, these ambiphilic species combine filled and vacant orbitals to break bonds of substrates by synergic donation and acceptance of electron density. However, merely binding and breaking bonds of a substrate has limited utility; the ability to form and liberate a product, while also regenerating the active reagent and thus making the process catalytic (or at least recyclable), is the true goal of small-molecule activation. It is here where very inert small molecules (e.g., N_2_, alkanes, etc.) present a major challenge to catalysis: the more inert the substrate, the more reactive the activating agent needs to be, and regenerating this high-energy species for catalytic turnover can become difficult in a thermodynamic (if not also kinetic) sense.

**Fig. 1:**
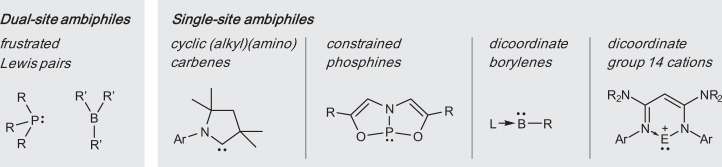
Selected representative main-group species capable of activating inert small molecules. R, R’: organyl substituent, Ar: aryl substituent, L: neutral donor unit, E: Si, Ge, Sn.

Our recent discovery of the binding and reduction of N_2_ at boron^11^ has provided inspiration for the use of main-group species to activate the most inert of substrates. Our work in this area not only highlights the potential of main-group single-site ambiphiles to perform very challenging transformations that are usually the preserve of transition metal chemistry, but has also uncovered unique aspects of the chemistry of main-group ‘metallomimetics’^9^. Of particular note here is that redox events occuring in our dinitrogen-borylene complexes seem to affect the N_2_ ligand much more directly than in dinitrogen complexes of transition metals, wherein oxidation and reduction tend to occur in nonbonding d orbitals of the metal and affect the bound N_2_ to a lesser extent. As a consequence, we were subsequently able to show that borylene species can mediate the reductive coupling of dinitrogen, a reaction that is known to occur under the harsh conditions of the upper ionosphere, but is not known in the coordination sphere of transition metals^12^.

From the point of view of catalysis, however, these findings solve only part of the problem, as the species responsible for activating N_2_ is thought to be a transient, high-energy borylene species of the form [(CAAC)BAr] (e.g. **A**, Fig. 2; CAAC = 1-(2,6-diisopropylphenyl)-3,3,5,5-tetramethylpyrrolidin-2-ylidene, Ar = Dur = 2,3,5,6-tetramethylphenyl). While this transient species was designed in order to maximize its interaction with and ability to bind dinitrogen, our very recent report of a one-pot, room-temperature synthesis of ammonium chloride from N_2_ using this system highlights a limitation of this approach. Indeed, while we were able to demonstrate the unprecedented synthesis of ammonium using a p-block species, along with a full elucidation and structural authentication of the key intermediates in the process (Fig. 2), a strong acid (HCl) is required in order to release the product from the boron reagent^13^. While this acidolysis was efficient in generating ammonium chloride from the system, it does not regenerate a catalytically active species, instead causing the decomposition of the boron-containing moiety. Indeed, the key intermediate [(CAACH)B(=NH_2_)(Dur)] (**B**, Fig. 2) in the demonstrated production of ammonium, while conceptually an adduct of NH_3_ with the presumed N_2_-activating species **A**, is essentially a conventional aminoborane. As such, **B** is considerably more stable than **A**, making the regeneration of the latter an energetically uphill process.

**Fig. 2:**
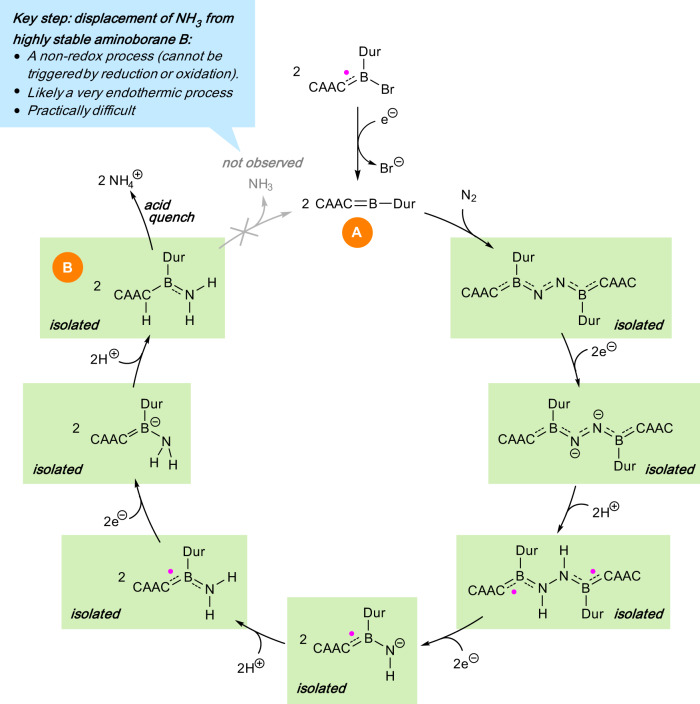
The borylene-mediated conversion of dinitrogen to ammonium. The key challenge in this reaction (highlighted by blue box) is the regeneration of the high-energy activating species **A** from highly stable pro-ammonia complex **B**. Details of the isolated compounds can be found in refs. ^11,13^. Figure adapted with permission from ref. ^13^, Springer Nature.

Thus, this new report^13^ provides hope for the future but also demonstrates the formidable challenge remaining to make the process practical, feasible and economical—namely the regeneration of a species that will activate dinitrogen, presumably a high-energy species such as **A** (Fig. 2). Research into transition-metal-free catalytic systems for ammonia synthesis will have to employ a different design philosophy than the one that led to dinitrogen reduction using **A**: while this species was engineered to maximize substrate (N_2_) binding, a functional catalyst will need to take into account end-product release. Hence, in our case, future work will focus on designing thermodynamically more stable borylenes in order to bind and reduce dinitrogen.

With this approach, we allow our main-group chemistry to be influenced by the established wisdom of transition metal catalysis. Indeed, the challenges of binding an inert substrate and subsequently releasing the product(s) are encompassed within the Sabatier principle^14^, which formulates that for catalytic efficiency, the interaction between reactants and catalysts should be neither too strong nor too weak. The time has come for main-group metallomimetics to follow the same logic, where fine-tuning may allow these species to go beyond the proof-of-concept of substrate binding and enter the field of efficient catalysis.

Thus, we persist undeterred towards the completion of the N_2_ conversion with our systems. In addition to the design of new borylenes with enhanced stability, there are a number of exciting future possibilities to explore, such as: (a) (photo)physical stimulus to displace the NH_3_ from **B**, such as photolysis or ultra-high vacuum, (b) the introduction of hemilabile donor units that can displace NH_3_ from **B** while retaining the “superambiphilic” characteristics of the borylene, (c) the introduction of auxiliary groups that can alter the tendency of the molecule to release the NH_3_ in a “switching” mechanism using, for instance, electrochemical stimuli, and (d) use of unsaturated NH_3_-accepting additives such as olefins, potentially effecting an overall olefin hydroamination process and the synthesis of amines.

Beyond our N_2_-activating system, one can envisage a number of ways to potentially develop a practical process for TM-free activation and catalysis of small molecules. Rational catalyst design strategies will be a natural starting point, e.g. enhancing the activating ability of a dual- or multi-site ambiphile such as an FLP, or tempering a single-site ambiphile such that the activating species is more energetically accessible. An alternative, but potentially complementary, strategy is to devise and test potential additives or physical methods that can assist with key steps of the reaction but will not quench the highly reactive activating species, such as photolysis, steric shielding, solubility differences, etc. An interesting example of this can be seen in our one-pot synthesis of ammonium chloride from N_2_^13^, wherein the strong reductant (potassium graphite) and the proton source (boric acid) coexist in the reaction mixture and take part in the reaction, but do not interact with each other due to their low solubility. In time, through catalyst design and the employment of additives and/or physical stimulus, there is hope that the challenge of combining high activating power with catalytic turnover can be met.

## References

[1] Stephan DW (2015). Frustrated Lewis Pairs: From Concept to Catalysis. Acc. Chem. Res..

[2] Qin Y, Zhu LH, Luo SZ (2017). Organocatalysis in inert C-H bond functionalization. Chem. Rev..

[3] Stephan DW (2016). The broadening reach of frustrated Lewis pair chemistry. Science.

[4] Martin D, Soleilhavoup M, Bertrand G (2011). Stable singlet carbenes as mimics for transition metal centers. Chem. Sci..

[5] Soleilhavoup M, Bertrand G (2020). Stable carbenes, nitrenes, phosphinidenes, and borylenes: past and future. Chem.

[6] Nesterov V (2018). NHCs in main group chemistry. Chem. Rev..

[7] McCarthy SM (2014). Intermolecular N−H oxidative addition of ammonia, alkylamines, and arylamines to a planar σ^3^-phosphorus compound via an entropy-controlled electrophilic mechanism. J. Am. Chem. Soc..

[8] Soleilhavoup M, Bertrand G (2017). Borylenes: an emerging class of compounds. Angew. Chem., Int. Ed..

[9] Légare M-A, Pranckevicius C, Braunschweig H (2019). Metallomimetic chemistry of boron. Chem. Rev..

[10] Do DCH (2020). N–H cleavage vs. Werner complex formation: reactivity of cationic group 14 tetrelenes towards amines. Chem. Commun..

[11] Légaré M-A (2018). Nitrogen fixation and reduction at boron. Science.

[12] Légaré M-A (2019). The reductive coupling of dinitrogen. Science.

[13] Légaré, M.-A. et al. One-pot, room-temperature conversion of dinitrogen to ammonia at a main-group element. *Nat. Chem*. 10.1038/s41557-020-0520-6 (2020). In press. 10.1038/s41557-020-0520-6 32929247

[14] Che M (2013). Nobel Prize in chemistry 1912 to Sabatier: Organic chemistry or catalysis?. Catal. Today.

